# Bibliometric analysis of Alzheimer's and dementia research in Latin America

**DOI:** 10.1002/alz.71395

**Published:** 2026-04-29

**Authors:** Mauricio Vazquez‐Guajardo, Alberto J. Mimenza‐Alvarado, Carlos Alejandro Martinez‐Zamora, Angel Lee, Oscar Abraham Jose Padilla Solis, José Francisco Parodi, Nilton Santos Custodio Capuñay, Sara G. Aguilar‐Navarro

**Affiliations:** ^1^ Geriatric Medicine and Neurology Fellowship Program, Instituto Nacional de Ciencias Médicas y Nutrición Salvador Zubirán, Tlalpan Ciudad de México Mexico; ^2^ Department of Geriatrics Instituto Nacional de Ciencias Médicas y Nutrición Salvador Zubirán, Tlalpan Ciudad de México Mexico; ^3^ Hospital Angeles Acoxpa, Tlalpan Ciudad de México Mexico; ^4^ Unidad de Inteligencia en Salud Publica Instituto Nacional de Salud Publica Cuernavaca Morelos Mexico; ^5^ Intellimedia Universidad de Guanajuato León de los Aldama Guanajuato Mexico; ^6^ Universidad de San Martin de Porres Facultad de Medicina Humana: La Molina Lima Peru; ^7^ Universidad Nacional Federico Villareal Lima Peru

**Keywords:** Alzheimer's disease, bibliometric analysis, dementia, Latin America and the Caribbean, research trends

## Abstract

**INTRODUCTION:**

Dementia is increasing rapidly in Latin America and the Caribbean (LAC), but research output remains limited. Tracking publication trends, themes, and collaborations is key to guiding regional research and policy.

**METHODS:**

Bibliometric analysis was conducted on dementia‐related publications from 21 LAC countries (1990 to 2024) using Scopus. Thirteen keywords identified relevant articles, classified into themes through artificial intelligence (AI)‐assisted and manual review. Bibliometrix and VOSviewer assessed publication trends, country and institutional output, and collaboration networks.

**RESULTS:**

Of 201,939 worldwide publications, 6003 (3%) included at least one LAC‐affiliated author. Brazil produced 49.9% of all dementia publications, followed by Argentina and Mexico. Clinical scenarios (15%) and basic science (14%) dominated thematic output. Mexico, Argentina, and Chile led regional collaboration efforts.

**DISCUSSION:**

Despite growth, dementia research in LAC remains concentrated in a few countries, with major thematic gaps and uneven collaboration. Strengthening cross‐country partnerships, broadening research themes, and increasing investment in applied and policy‐focused studies are essential.

## Highlights

- ADRD publications in LAC increased but remained only 3% of global output.
- AD accounted for 61% of LAC dementia‐related publications.
- Brazil produced 49.9% of all LAC dementia publications from 1990 to 2024.
- Mexico, Argentina, and Chile led regional collaboration efforts.
- Clinical scenarios (15%) and basic science (14%) were the top research themes.

## 1 INTRODUCCION

The prevalence of Alzheimer's disease and related dementias (ADRD) is rising rapidly in Latin America and the Caribbean (LAC), driven by a major demographic shift marked by accelerated population aging.^1^ As of April 2025, the population of LAC represents approximately 8.11% of the global population and is projected to experience a four‐fold increase in the number of people living with dementia between 2015 and 2050.^2^, ^3^

Notably, this demographic transition is occurring at a significantly faster pace in LAC compared to high‐income countries (HICs).^4^ Two out of three people with dementia live in low‐income and middle‐income countries (LMICs).^5^ Despite the anticipated surge in ADRD cases, scientific research output in the region remains disproportionately low. LAC continues to be underrepresented in global ADRD research initiatives, including large‐scale clinical trials, limiting the applicability of international findings to local populations. This mismatch between the growing disease burden and the current level of research activity underscores the urgent need for a paradigm shift in how ADRD is studied and addressed in LAC.

Encouragingly, the last two decades have witnessed meaningful progress. Investment in dementia research in the region has increased, with the Alzheimer's Association currently funding 41 active research projects across Latin America, totaling over US$4 million. In recent years, interactions between researchers from high‐income countries and LMICs have increased but have largely followed a neocolonial model with little benefit for LMIC researchers.^6^ This represents a four‐fold increase in funding compared to previous decades and reflects a growing recognition of the importance of locally driven research.^7^

Nonetheless, while these developments mark important progress, the region still lags far behind HICs in terms of infrastructure, publication output, and inclusion in global research collaborations. Barriers such as limited funding opportunities, insufficient local capacity, and structural inequalities continue to hinder progress, a challenge amplified by the immense diversity across LAC in histories, cultures, political systems, and healthcare structures.^6^ Addressing these gaps is essential not only for improving care and outcomes in LAC but also for enriching global understanding of ADRD through more inclusive and diverse research.

In this study, we conducted a comprehensive bibliometric analysis of dementia‐related publications from 21 LAC countries spanning the period from 1990 to 2024. By examining publication trends, thematic focus, and research collaborations, this analysis aims to quantify the region's scientific output, highlight existing gaps, and assess the extent of recent investments in dementia research. Through this data‐driven approach, we seek to provide an evidence‐based foundation for future strategies to strengthen and expand ADRD research in LAC.

## 2 METHODS

### 2.1 Data source and search strategy

A comprehensive literature search was conducted in the Scopus database to identify relevant publications on dementia research in Latin America. It was decided to use Scopus exclusively, not only because it is an appropriate source for citation tracking but also because many LAC journals are not indexed in PubMed but are included in Scopus. This database offers comprehensive coverage across geographical regions, journals, and subject areas, along with robust data integrity. The search was initiated from January 1990 and concluded in October 2024. It was decided to start in 1990 after the widespread adoption of the first clinical criteria of AD.^8^ It was limited to 21 LAC countries: Argentina, Bolivia, Brazil, Chile, Colombia, Costa Rica, Cuba, Dominican Republic, Ecuador, El Salvador, Guatemala, Haiti, Honduras, Mexico, Nicaragua, Panama, Paraguay, Peru, Puerto Rico, Uruguay, and Venezuela.

**RESEARCH IN CONTEXT**

1. **Systematic review**: We conducted a comprehensive bibliometric analysis of dementia‐related publications from 21 LAC countries using Scopus (1990 to 2024). Keyword selection combined expert consensus and AI‐assisted refinement to capture major dementia syndromes. Publications were screened, deduplicated, and thematically classified into 16 categories using AI‐assisted and manual review. Collaboration networks and productivity metrics were examined using Bibliometrix and VOSviewer.
2. **Interpretation**: Our findings demonstrate growth in ADRD research in LAC countries but reveal marked geographic and thematic disparities. Research output is heavily concentrated in a few countries, primarily Brazil, and dominated by clinical and basic science topics, whereas other relevant topics remain underrepresented. Collaboration networks show increasing connectivity but persistent structural imbalances.
3. **Future directions**: Future research should prioritize expanding studies on non‐Alzheimer's dementias, strengthening regional consortia and equitable cross‐country collaborations, increasing applied research on care models, guidelines, and public health, and developing locally relevant prevention and risk‐reduction strategies.

A meticulous keyword selection process was employed to ensure optimal search sensitivity and specificity. A combination of expert consensus (geriatricians and neurologists) and artificial intelligence (AI) was utilized to identify the most relevant keywords. We used platforms like Gemini Deep Research and ChatGPT. The final set of 13 keywords included dementia, mild cognitive impairment, Alzheimer's disease, frontotemporal lobar degeneration, Lewy body, Creutzfeldt‐Jakob, vascular cognitive impairment, posterior cortical atrophy (PCA), progressive supranuclear palsy, multiple system atrophy, Wernicke‐Korsakoff, primary progressive aphasia (PPA), and limbic‐predominant age‐related encephalopathy. The following information was extracted from the identified publications: author(s), document title, year, source title, volume, issue, pages, citation count, source and document type, publication stage, digital object identifier (DOI), open access status, affiliations, PubMed ID, publisher, editor(s), language of the original document, correspondence address, abbreviated source title, abstract, author keywords, and indexed keywords.

To ensure data quality and consistency, duplicate records were removed. Additionally, the following document types were included in the analysis: article, book, book chapter, conference paper, data paper, editorial, and short survey.

**FIGURE 1:**
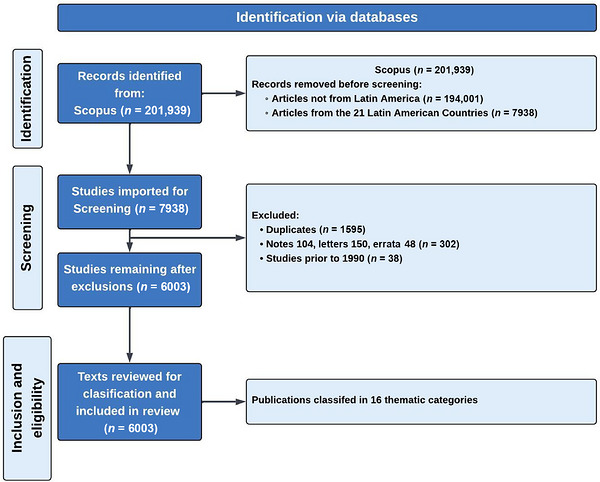
PRISMA flow diagram of the literature screening and inclusion process. Starting from 201,939 records identified in Scopus, articles were filtered for relevance to Latin America (*n* = 7938). After removing duplicates (*n* = 1595) and other exclusions (*n* = 388), 6003 texts were included in the final review and classified by publication category and subcategory.

A PRISMA flow diagram was constructed to visually represent the screening process and the number of studies included at each stage of the review (Figure 1).

### 2.2 Article classification and data analysis

We then classified the publications by country of origin and by theme. Using AI, we first performed a preliminary classification of the articles into the 16 thematic categories (pathology/basic sciences, genetics, epidemiology, prevention, socioeconomics, symptomatology, cognitive testing, diagnostic approach, guidelines, imaging, fluid biomarkers, pharmacological, non‐pharmacological, quality of life, and palliative care). This was achieved by utilizing ChatGPT to generate a custom Visual Basic for Applications (VBA) macro for Microsoft Excel. The macro was designed to scan a defined column and change both the background color and font color of cells that contained specific keywords related to each thematic category (e.g. “donepezil” for the pharmacological category or “mice” for the basic science category). The macro used RGB color codes to highlight relevant cells, allowing for easy visual identification and counting of matching articles. This process was repeated for each of the 16 categories using category‐specific keywords. Articles that met the criteria for more than one category were reviewed and assigned to the most appropriate category based on thematic orientation. The remaining unclassified articles were manually reviewed and categorized by the lead author (MVG). Those that did not clearly fit into any of the predefined themes were labeled as “other.”

### 2.3 Publication trends, country‐wise contributions, and research collaborations

The retrieved dataset was downloaded from Scopus in.csv format and uploaded to Bibliometrix in R and VOSviewer to examine publication trends, country‐wise contributions, and research collaborations. To analyze journal productivity over time, we identified the most published LAC journals in dementia research and visualized their cumulative publication trends using Bibliometrix. The distribution of publications by country was assessed by calculating the percentage contribution of each nation, which was then represented in a choropleth map. To explore research collaborations, we used VOSviewer to construct a country‐collaboration network, using co‐authorship analysis at the country level with full counting. Under this approach, each country involved in a multicountry publication is counted once, node size represents publication volume, and links depict the relationships between countries, with thicker lines indicating stronger collaboration. A color gradient was applied to reflect the timeline of collaborative efforts. We also calculated the total link strength (TLS), which represents the sum of all collaborative links established by a given country with others. This bibliometric metric serves as an indicator of the centrality and influence of a node within the scientific collaboration network – whether an author, institution, or country. In this study, TLS was used to rank countries according to their level of international collaboration and connectivity within the publication network.^9^, ^10^

## 3 RESULTS

Of the 201,939 articles identified worldwide using the 13 keywords, 7938 included at least one co‐author affiliated with a Latin American institution. After removing 1595 duplicates, 302 notes, letters, and errata, and 38 articles published before 1990, a total of 6003 articles were retained for analysis (Figure 1). Among these, 5511 (91%) were original research articles or reviews, while the remaining publications comprised conference papers, book chapters, editorials, short surveys, books, and data papers.

The most frequent dementia‐related publications focused on Alzheimer's disease (61%) and dementia in general (25%). The remaining 14% covered a range of conditions, including mild cognitive impairment (4%), frontotemporal dementia (2%), rapidly progressive dementias (2%), and other less common forms such as vascular dementia, Lewy body/Parkinson's dementia (LBD), PPA, PCA, and HIV‐associated dementia (Table 1).

**TABLE 1:** Publication trends on types of dementia. This table shows the distribution of scientific publications across various dementia types. Research is overwhelmingly focused on Alzheimer's disease, which accounts for 61% (3669 publications) of the literature. Dementia (non‐specified) is the second largest, representing 25% (1512 publications).

Dementia subtype	Total (*n* = 6003)	Percentage (%)
Alzheimer's disease	3669	61%
Dementias (non‐specified)	1512	25%
Mild cognitive impairment	266	4%
Frontotemporal dementia	140	2%
Rapidly progresssive dementias (Creutzfeldt‐Jakob disease and others)	127	2%
Other atypical parkinsonisms	72	1%
Lewy body dementia and Parkinson's dementia	71	1%
Vascular dementia	64	1%
Primary progressive aphasia	52	1%
HIV‐associated dementia	16	0%
Posterior cortical atrophy	13	0%
Limbic‐predominant age‐related TDP‐43 encephalopathy (LATE)	1	0%

**FIGURE 2:**
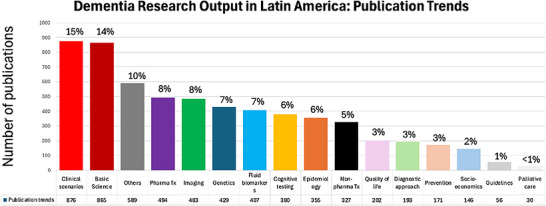
Distribution of dementia research publications in Latin America by category. The bar chart illustrates the number of publications (*y*‐axis) across 16 different research categories (*x*‐axis), showing both the absolute number and the percentage of the total. The most prevalent categories are “Clinical scenarios” (*n* = 876, 15%) and “Basic science” (*n* = 865, 14%). The least common category is “Palliative care” (*n* = 30, <1%). Pharma Tx (pharmaceutical treatment); non‐pharma Tx (non‐pharmaceutical treatment).

Thematic analysis of the research output revealed that “Clinical scenarios” and “Basic science” were the most prevalent research categories, accounting for 15% and 14% of all publications, respectively. These two areas, together with “Others,” represented the largest shares of research, totaling nearly 40% of the output. Following these, “Pharmacological treatment,” “Imaging,” “Genetics,” and “Fluid biomarkers” each made up 7% to 8% of the total publications, indicating a balanced focus on therapeutic and diagnostic research. Other categories such as “Cognitive testing,” “Epidemiology,” and “Non‐pharma treatment” comprised smaller but still substantial portions of the research, each making up 5% to 6% of the output. In contrast, themes related to “Socioeconomics,” “Guidelines,” and “Palliative care” represented the smallest fractions of the research, at 2% or less, suggesting these areas are currently underrepresented in the regional research landscape (Figure 2).

**FIGURE 3:**
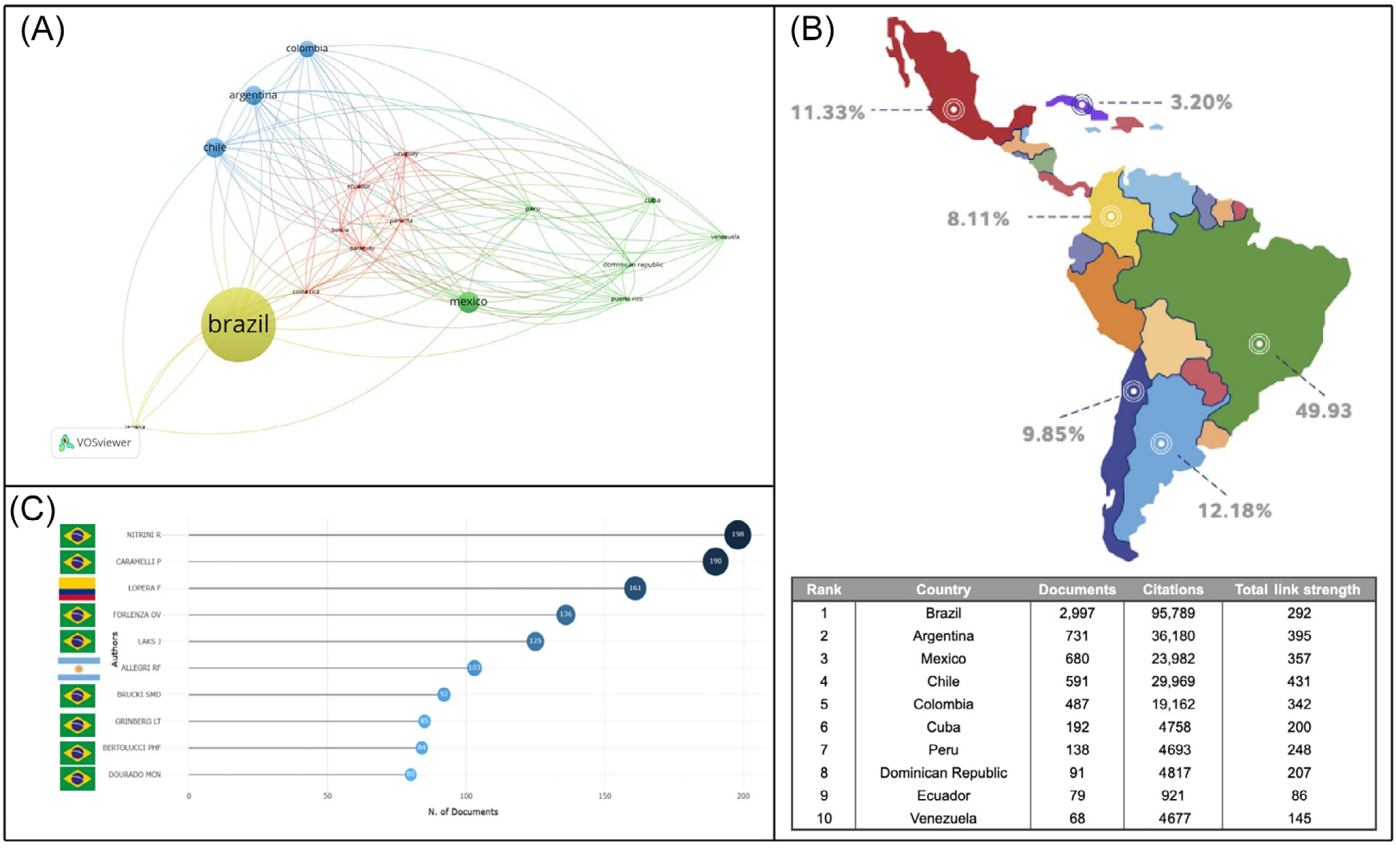
Country‐level productivity, impact, and collaboration in Latin American dementia research. The figure shows the co‐authorship network of country collaborations (A), geographic distribution of publications in Latin America and the Caribbean (B), ranking of the top 10 most‐cited authors (C), and a summary table ranking countries by documents, citations, and total link strength (TLS) (table, bottom right).

The country that published the most and had the most growth in recent decades was Brazil, with 49.93% of the publications. Following Brazil, the countries with the highest contributions were Argentina (12.18%), Mexico (11.33%), and Chile (9.85%). Other relevant contributors were Colombia, with 8.11%, and Cuba, with 3.2%. This leadership in publication volume is mirrored by individual researcher impact; of the top 10 most‐cited authors, eight are from Brazil, with one from Argentina and one from Colombia completing the list. In the country‐network analysis, Brazil, Argentina, Chile, Colombia, and Mexico emerged as the largest nodes, reflecting their higher publication volumes. The network visualization shows that Brazil acts as a central hub, with strong collaborative ties particularly to Argentina, Chile, Colombia, and Mexico. The thickness of the connecting lines denotes the intensity of co‐authorship, with the strongest collaborations observed between Brazil and the Southern Cone countries. The network is also organized into regional clusters; for instance, Cuba and the Dominican Republic show strong links with Mexico and Venezuela. In contrast, smaller nodes such as Guatemala, El Salvador, Paraguay, and Bolivia exhibit fewer and weaker connections within the network (Figure 3).

To quantify the level of international collaboration, the TLS was calculated for each country. This bibliometric parameter represents the sum of all collaborative links and serves as an indicator of a node's centrality and influence. According to this metric, the most connected countries are Chile (TLS 431), Argentina (TLS 395), Mexico (TLS 357), and Colombia (TLS 342). Notably, while Brazil has the highest number of documents and citations, its TLS (292) is lower than that of several other regional leaders, suggesting that while it is highly productive, other countries have a greater overall strength of collaboration across the network. The lower TLS values for countries like Ecuador (86) and Venezuela (145) quantitatively confirm their more peripheral positions in the network visualization.

**FIGURE 4:**
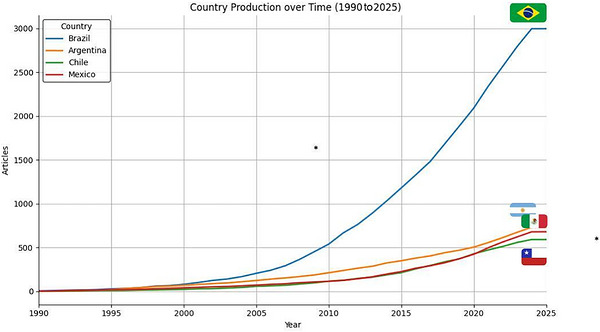
Growth of scientifc production on dementia over time (1990 to 2024) for the top four contributing countries. The line graph plots the cumulative number of articles (*y*‐axis) published over the 34‐year period (*x*‐axis) for Brazil, Argentina, Chile, and Mexico. The chart highlights Brazil's significant and accelerating lead in publication volume, accumulating over 3500 articles. The other three countries show similar, though more modest, growth trajectories, each reaching approximately 1000 publications by 2024.

Over the last 34 years, the scientific production of dementia‐related articles in Latin America has grown substantially. In 1990, publications were minimal across the region, but by 2024 the numbers increased markedly. Brazil stands out with more than 3000 articles, positioning itself as the leading contributor in the region. Notably, since 2020 Brazil has pulled ahead more clearly compared with other countries, widening the gap in scientific output. Meanwhile, other countries such as Mexico, Argentina, and Chile also demonstrated significant growth, each producing close to 1000 publications (Figure 4).

**FIGURE 5:**
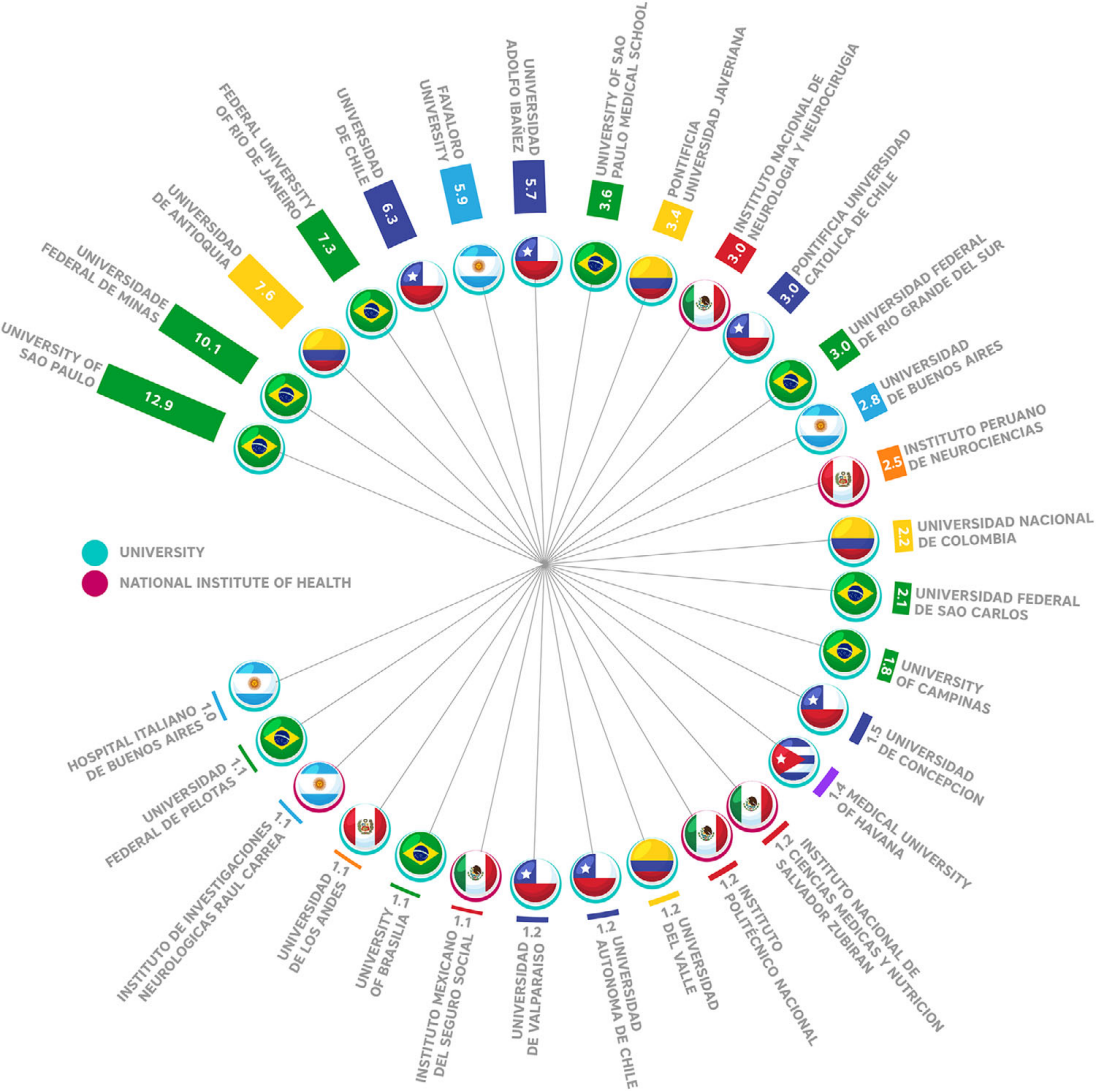
Leading universities and health institutes in Latin American dementia research by publication output. The radial bar chart ranks the top institutions based on their percentage share of the total publications, where the length of each bar and corresponding number represent the institution's percentage contribution. Institutions are ranked in descending order, with each bar also displaying the corresponding country's flag. Universities are shown with a blue circle around the flag and health institutes with a pink circle.

The analysis of institutional contributions to dementia‐related research in Latin America revealed a marked predominance of Brazilian universities. The University of São Paulo accounted for the highest share of publications (12.9%), followed by the Federal University of Minas Gerais (10.1%) and the Federal University of Rio de Janeiro (7.6%). Other institutions with notable participation included the Universidad de Antioquia in Colombia (7.3%) and the Universidad de Chile (6.3%). Additional contributions were observed from universities in Argentina, Mexico, Peru, Cuba, and other South American countries, each ranging between 1% and 5% of the total output (Figure 5).

**FIGURE 6:**
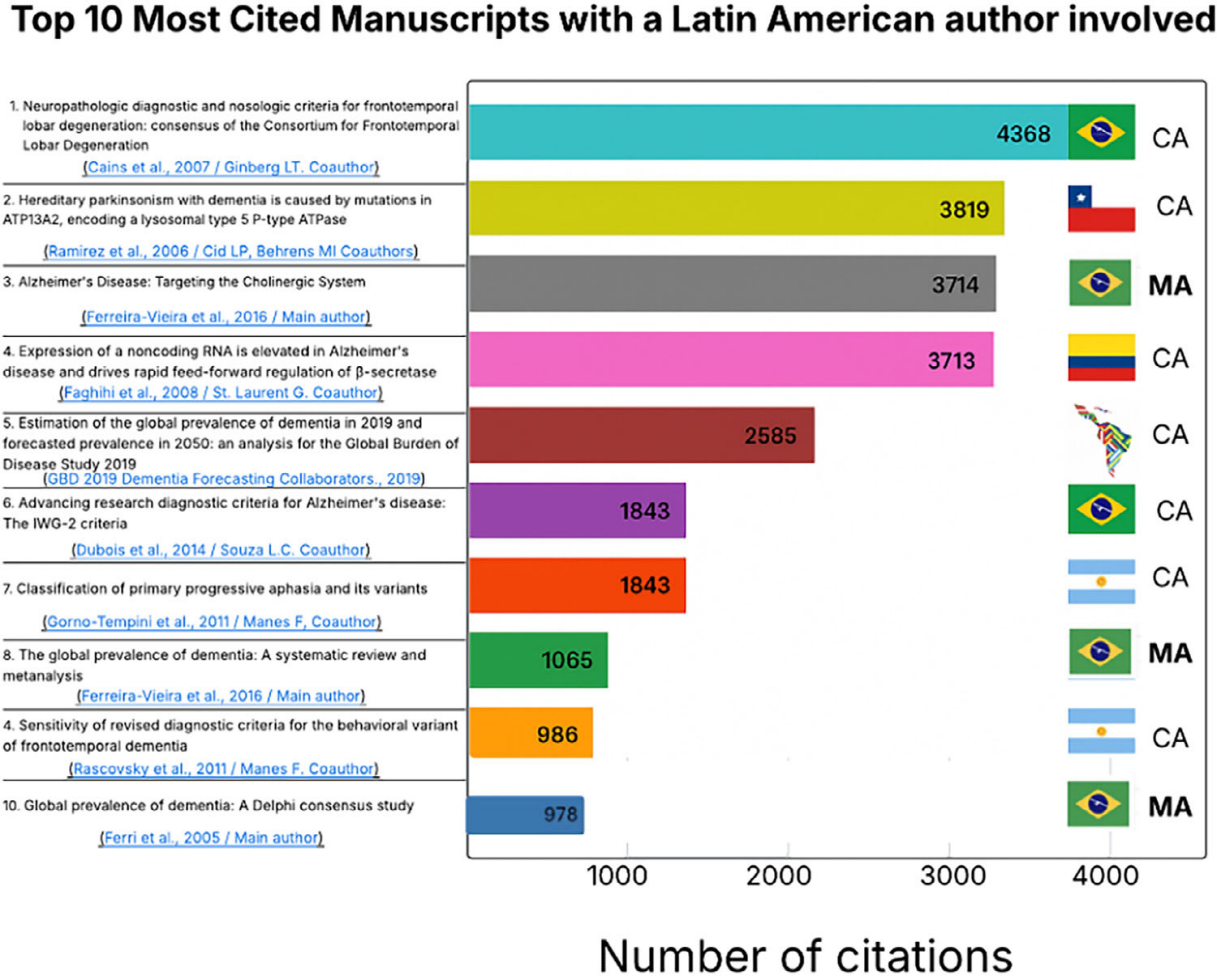
The 10 most cited manuscripts involving a Latin American author, ranked by citation count. This horizontal bar chart displays the absolute number of citations for the top 10 articles. The country of the Latin American author (e.g., Brazil, Chile, Argentina, Colombia) and their role (CA: co‐author; MA: main author) are indicated next to the bar.

Figure 6 presents the top 10 most cited manuscripts. Among these highly cited works, two focused on genetics, four on clinical criteria, three on epidemiology, and one on pharmacology. Notably, only three of these publications had Latin American researchers as main authors, all from Brazil, while the remaining seven involved Latin American scientists as co‐authors, representing Colombia, Chile, Argentina, and Brazil. The number of citations varied widely, with the most influential article reaching 4368 citations, whereas the least cited in this group still achieved a significant 978 citations (Figure 6).

## 4 DISCUSSION

In this bibliometric review, encompassing ADRD publications from 1990 to 2024, we examined publication trends, thematic focus areas, and research collaborations among LAC countries using a data‐driven approach. Our findings confirm that, despite a steady increase in ADRD research output, there is a marked disparity in publication volume among countries, and several important research topics – such as non‐Alzheimer's dementias – remain underrepresented in the region.

Of the 201,939 ADRD‐related articles published worldwide between 1990 and 2024, only 6003 included at least one author affiliated with a LAC institution, representing a mere 3% of the global total. In contrast, during the same period, the United States produced 112,110 publications, accounting for approximately 56% of all ADRD research globally. This contrasts with dementia prevalence in LAC, which is among the highest in the world, with estimates ranging from 8% to over 14% in people aged 60+.

Our bibliometric analysis reveals a strong geographic concentration of dementia research within a handful of LAC countries. Brazil accounts for nearly half of all regional publications, followed by Argentina, Mexico, Chile, Colombia, and Cuba. This Brazilian dominance aligns with previous bibliometric studies showing a steep rise in national output over the past two decades and is reflected in institutional productivity, led by the University of São Paulo, Federal University of Minas Gerais, and Federal University of Rio de Janeiro.^11^ The marked leadership of Brazil in dementia research should be interpreted within the broader context of structural heterogeneity across LAC. Brazil is not only the largest country in the region but also the most populous, with over 210 million inhabitants, which naturally expands its academic base, patient population, and research workforce.^12^ However, population size alone does not fully explain its scientific output. Over the last two decades, Brazil has implemented sustained national policies to strengthen science and innovation, including the expansion of federal universities and the consolidation of funding agencies such as CAPES and Brazilian National Council for Scientific and Technological Development (CNPq), which have promoted graduate training, international collaboration, and large multicenter projects.^13^ In addition, Brazil consistently allocates a higher proportion of public resources to research and development than most countries in the region, historically around 1% to 1.3% of gross domestic product (GDP), whereas many Latin American countries invest below 0.5%.^14^ This sustained funding has fostered stronger research institutions, networks, and publication output. Brazil also has the largest research workforce in the region, with 6.38 full‐time equivalent researchers per 1000 employed persons, far exceeding most LAC countries.^15^, ^16^

As in the rest of the world, AD dominates dementia‐related publications. In LAC, the high prevalence and societal burden of AD have attracted significant research funding and public interest, leading to the establishment of large research consortia, longitudinal studies, and clinical trials.^7^ In contrast, other forms of dementia, notably vascular dementia and LBD, remain underrepresented despite their recognized clinical importance.^17^ Vascular dementia is one of the leading causes of dementia and disability globally and in LAC, where vascular risk factors such as hypertension, diabetes, and obesity are highly prevalent. However, limited access to neuroimaging and standardized diagnostic tools, along with underdiagnosis in clinical and population studies, contributes to the scarcity of vascular dementia research.^2^, ^17^, ^18^ Similarly, LBD – often underrecognized and misclassified as Parkinson's or Alzheimer's disease – faces diagnostic and research challenges that hinder publication output.^19^ Moreover, constrained research capacity, fragmented collaboration, and funding priorities centered on AD biomarkers and therapies further explain this imbalance.

In our study, we categorized dementia‐related publications from LAC into 16 thematic areas. The most frequent topics were clinical scenarios (15%) and basic science (14%), which largely comprised small case reports, case series, or exploratory laboratory studies with limited translational application. In contrast, less than 10% of publications addressed quality of life, diagnostic approaches, prevention, or socioeconomic factors, and almost none focused on guidelines or palliative care, indicating important gaps in applied and community‐oriented research. This thematic distribution underscores a research landscape heavily oriented toward clinical description and biological mechanisms, rather than toward public health, care models, or policy development.

When compared to global and regional bibliometric analyses, this imbalance becomes more evident. In HICs, dementia research has progressively diversified to include domains such as care delivery, quality of life, prevention, and public health.^20^ International frameworks, such as the World Health Organization's Global Action Plan on Dementia, and collaborative research consortia have prioritized risk reduction, caregiving support, and health system preparedness, alongside ongoing advances in biomarker discovery and pharmacological innovation.^21^, ^22^ These regions also produce a greater volume of research on clinical guidelines, policy implementation, and palliative care, reflecting the maturity of their healthcare systems and the existence of national dementia strategies.

In contrast, LMICs, including those in LAC, continue to show a narrower research focus. Prior analyses described a predominance of clinical and basic science investigations, with scarce attention being paid to care models, prevention, or social determinants of health.^5^ Although initiatives such as the 10/66 Dementia Research Group have generated valuable epidemiological and social data within LAC, these efforts represent a small proportion of total regional output.^23^ Similar patterns are observed across other regions of the Global South, including Southeast Asia and sub‐Saharan Africa, where research priorities are often shaped by immediate clinical and diagnostic demands, limited funding, and the absence of integrated dementia policies.^24^

This bibliometric study has several strengths, including its comprehensive coverage of 21 LAC countries over 34 years, the use of Scopus to capture a wide range of regional journals, and a rigorous methodology combining expert input with AI‐assisted classification into 16 thematic areas. In addition, network analyses using VOSviewer and TLS metrics provided detailed insights into intercountry and institutional collaborations, allowing identification of structural patterns and research gaps. However, the study also has limitations. Reliance on a single database may have excluded some regional publications, and despite careful keyword selection, relevant studies might have been missed or misclassified. Although we implemented a meticulous, expert‐informed keyword selection strategy, the finite number and scope of search terms remain a limitation, as variations in terminology and indexing practices across countries may have led to the omission of relevant publications not captured by the predefined terms. Moreover, bibliometric analyses primarily quantify output and collaborations without assessing research quality or clinical impact, and the assignment of thematic categories, even with manual review, may introduce some subjective bias. Finally, citation‐based metrics may underrepresent recent publications, potentially skewing influence measures toward older studies.

In conclusion, dementia research in LAC has grown substantially over the past three decades, yet it remains concentrated in a few countries, with Brazil leading regional output. While clinical and basic science studies dominate, critical areas such as vascular dementia, LBD, prevention, care models, socioeconomics, guidelines, and palliative care remain underrepresented. Research collaborations are expanding, but structural imbalances persist, with smaller institutions and countries remaining on the periphery of the scientific network. These findings highlight both the progress achieved and the gaps that need to be addressed, underscoring the importance of fostering regional consortia, enhancing cross‐country partnerships, and investing in diverse, applied, and policy‐oriented dementia research to better meet the growing regional burden.

## CONFLICT OF INTEREST STATEMENT

The authors have no conflicts of interest to declare. Author disclosures are available in the supporting information.

## CONSENT STATEMENT

Consent was not necessary.

## Supporting information



Supporting Information
